# Positioning HIV SMRTcap within the HIV reservoir toolbox

**DOI:** 10.1371/journal.ppat.1014257

**Published:** 2026-05-29

**Authors:** Anthony Loddo, Pierre Delobel, Camille Vellas

**Affiliations:** 1 Univ‌‌ Toulouse, INSERM, CNRS, Infinity – Toulouse Institute for Infectious and Inflammatory Diseases, Toulouse, France; 2 Department of Infectious Diseases, Univ Toulouse, CHU Toulouse, Toulouse, France; 3 Laboratory of Virology, Univ Toulouse, CHU Toulouse, Toulouse, France; University of Iowa, UNITED STATES OF AMERICA

Antiretroviral therapy (ART) efficiently suppresses HIV replication but does not eliminate long-lived infected cells carrying HIV proviruses, which remain the major barrier for a cure. Recently, Sadri et al., presented a novel sequencing approach to characterize the HIV reservoir: HIV SMRTcap [1]. This newly developed technique expands the toolbox available by adding long-read sequencing approaches combined with provirus integration sites analysis previously missing. Here, we discuss how HIV SMRTcap should be positioned within the current reservoir toolbox, focusing on the biological dimensions it captures, its main strengths and limitations and its implications for HIV cure research.

## Measuring the HIV reservoir: Beyond a single measurement

The HIV reservoir, or proviral reservoir, can be defined at different levels depending on the perspective used. These include the organ level, the cellular level, the molecular level (the integrity of the provirus), but also the dynamic level (how proviruses persist over time, through clonal proliferation or ongoing replication) [2]. Even in people living with HIV (PLWH) under ART with sustained viral suppression, the proviral reservoir remains complex and dynamic [3]. This multidimensionality is central to the interpretation of reservoir assays: each method captures only part of the reservoir and should be positioned according to the biological information it provides.

Quantitative PCR, digital PCR [4], and the Intact Proviral DNA Assay (IPDA) address the question of “how much proviral DNA is present”. Additionaly, IPDA enables the quantification of likely intact proviruses, 3′-deleted and/or hypermutated proviruses and 5′-deleted proviruses [5,6]. Its strengths are scalability and suitability for large cohort studies. However, IPDA only provides an estimation of proviral integrity based on targeted regions and does not give access to the full proviral sequence, or information on clonality.

To address the question of “what is integrated” and to determine whether proviruses are truly intact, sequencing-based approaches such as near-full-length proviral sequencing (nFGS) and full-length individual proviral sequencing (FLIP-seq) have been developed [7,8]. These methods can be achieved on blood [9] and tissue samples [10] and provide detailed information on proviral intactness, large deletions and APOBEC3G/3F-induced hypermutations. However, they may be affected by amplification bias, as only approximately 30% of intact proviruses are detected, while proviruses with large internal deletions are detected at expected frequencies [6].

A third dimension is functional competence as intactness does not necessarily imply that a provirus can produce infectious virus. This question is addressed by quantitative viral outgrowth assays (QVOA), and related induction-based approaches [11]. QVOA remains the reference method to measure the inducible, replication-competent reservoir. However, it is labor-intensive, requires large numbers of viable cells, and is therefore not suitable for tissues analyses. It is also known to underestimate reservoir size, as not all intact proviruses are inducible under experimental conditions and may require repeat stimulation [9].

Beyond structure and function, the chromosomal context of proviral integration represents another key dimension of HIV reservoir. Mapping integration sites addresses a complementary question: “where in the host genome is the provirus located?”. Widely used approaches include ligation-mediated-PCR (LM-PCR) [12] and integration site loop amplification (ISLA) [13]. These methods allow identification of integration sites, within active or inactive genes and enable the detection of clonal expansions. However, they lack direct linkage to proviral sequence integrity, are technically complex, and face challenges when integrations occur in repetitive genomic regions.

An elegant strategy to associate proviral sequence and integration site information relies on limiting dilution, in which each well contains a single provirus, followed by splitting the material for separate proviral sequencing and integration site analysis [14,15]. While powerful, this approach is labor-intensive and not compatible with large cohort studies. This is the practical niche in which HIV SMRTcap becomes valuable: it uses long-read capture-based sequencing to recover both proviral sequence and integration site information within the same workflow, without requiring single-provirus limiting dilution.

## HIV SMRTcap: Linking proviral sequence and integration site information: Strengths and limitations

Long-read sequencing approaches aim to integrate proviral sequence and chromosomal location in a single assay. HIV SMRTcap, recently described by Sadri and colleagues, achieves this by combining targeted hybridization capture (cap) with single-molecule real-time (SMRT) sequencing [1]. This approach enables molecular-level clonality analysis, sample multiplexing, and offers a view of the viral landscape. This innovative and powerful method has been validated by the authors across different HIV subtypes, in different cell types and tissues, and compared with established reservoir assays (IPDA, QVOA, LM-PCR, and FLIP-seq).

Compared with the IPDA, HIV SMRTcap’s main added value lies in the molecular information it provides on proviral sequences and integration sites. Conversely, unlike the IPDA, it is not primarily designed as a scalable quantification assay. There may be discrepancies between the HIV SMRTcap and IPDA regarding the classification of provirus intactness. In the study by Sadri and colleagues, some proviruses detected as intact by the IPDA, as their 5′ and 3′ targeted regions are preserved, were classified as defective by HIV SMRTcap due to internal deletions affecting accessory genes *vif*, *vpr*, and *vpu*. This discrepancy illustrates an important interpretative point. The absence, or low recovery of intact proviruses by HIV SMRTcap may reflect their true rarity, but may also result from technical and sampling factors, including limited input material, amplification bias, capture efficiency, or DNA shearing. Moreover, although proviruses with accessory gene deletions are generally classified as defective, some of them may be replication competent because such deletions do not always prevent protein production and replicative capacity [16,17].

**Fig 1:**
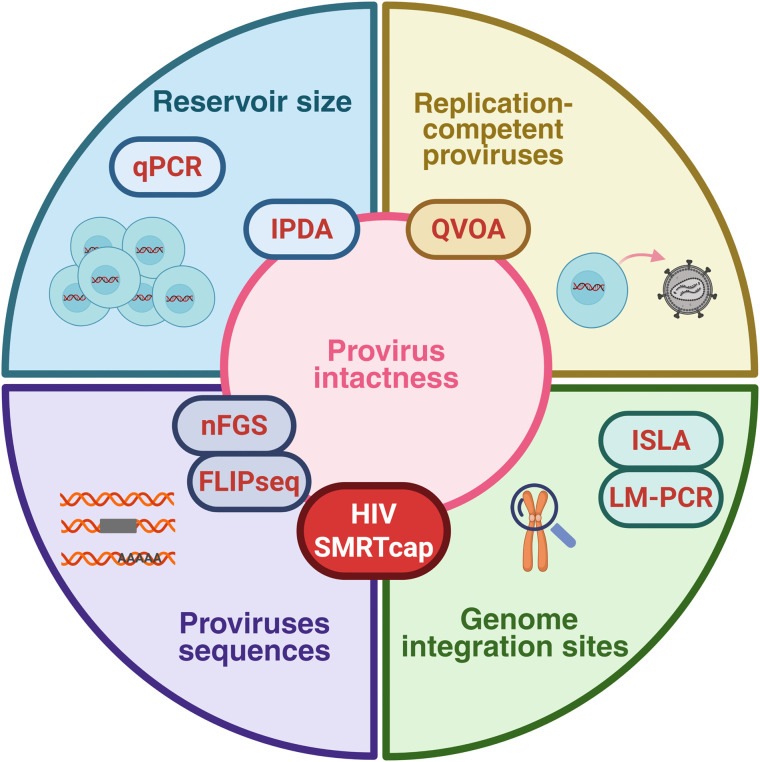
Positioning HIV SMRTcap within the HIV reservoir toolbox. Biological dimensions of the HIV reservoir captured by different assays are represented. Proviral intactness can be assessed using IPDA, QVOA, nFGS, FLIP-seq, and HIV SMRTcap. HIV SMRTcap bridges proviral sequence and chromosomal integration within a single assay, but does not directly assess reservoir size or functional competence. Created in BioRender. Vellas, C. (2026) https://BioRender.com/ztp0rqq.

Compared with nFGS or FLIP-seq, HIV SMRTcap provides additional information by linking proviral sequence to chromosomal integration site. This is a major strength of the long-read strategy, as it can recover viral and host-genomic information at the single-molecule level without requiring limiting-dilution approaches to isolate one provirus per well. This makes it particularly useful to explore HIV persistence within infected cells from patient samples. Repeated detection of the same proviral sequence at the same integration site can indicate clonal expansion of the provirus. However, the use of hybridization probes could also lead to the loss of hypermutated proviruses that are not efficiently captured, whereas they are detected in nFGS and FLIP-seq. While these proviruses are generally replication-incompetent, as argued by the authors, their exclusion should be kept in mind when studies aim to comprehensively characterize the full proviral reservoir or to study the clonality of defective proviruses. Another consideration is that, because HMW DNA is sheared prior to capture, provirus-containing fragments become highly diluted among a large excess of non-specific genomic fragments, leading to semi-stochastic sampling and reduced sensitivity at low proviral abundance. A main concern with the HIV SMRTcap approach is that it may deliver results that are inferior to Illumina-based viral sequencing obtained with limiting dilution techniques, due to biases introduced during amplification and sequencing. Fig 1 summarizes the position of HIV SMRTcap within the HIV reservoir toolbox.

The open-access bioinformatic pipeline accompanying HIV SMRTcap is an important strength of the assay, as it facilitates implementation and reproducibility. However, its availability does not fully remove the need for expertise in long-read sequencing analysis. Template switching events may occur, some sequences may require bioinformatically reassembled due to artefactual shearing, and appropriate quality control parameters must be carefully selected (e.g., HiFi CCS read number thresholds, exclusion of template switching events, signal-to-noise filtering). The bioinformatic definition of “intactness” used by the author (>75% of the 11 viral genes present) should ideally require a sequence verification in some cases.

Practical implementation is an important consideration. HIV SMRTcap requires high-quality HMW DNA, hybridization capture probes, PacBio SMRT sequencing, and long-read bioinformatic expertise. These requirements may limit broad implementation in laboratories without direct access to long-read sequencing infrastructure. At the same time, the method has practical advantages as discussed above. Samples can be multiplexed, reducing sequencing costs and experimental workload. The workflow is more compatible with batch processing than approaches requiring multiple dilution plates per sample. In this sense, it may be more scalable for cohorts although its scalability will depend on local sequencing infrastructure.

## Implications for HIV cure research

No single assay can capture all dimensions of the HIV reservoir. Importantly, the choice of assay is strongly influenced by the biological material under study, with tissue samples containing limited numbers of cells, making it difficult to apply multiple assays. The HIV SMRTcap workflow still needs further evaluation in larger numbers of samples from PLWH with undetectable viral load, especially tissue samples. However, it may represent an adaptable assay for such sample types and cohort-based studies.

Integrated strategies have important implications for HIV cure research. They can refine our understanding of viral rebound and provirus persistence, and improve the interpretation of intervention studies. A key challenge moving forward is not simply to develop new technologies, but to optimize and scale existing ones so they can be applied across larger cohorts and diverse sample types. Harmonized frameworks will allow studies to be additive rather than parallel, strengthening collective progress in the field. To improve comparability and cumulative knowledge we propose minimal reporting standards across studies using HIV SMRTcap: input cell numbers and DNA quantification, explicit definitions of clonality, sequencing quality parameters, pipeline settings, quality-control thresholds used to identify artifacts, and open access to sequence data.
